# Adaptation and Evaluation of a Multi‐Component School‐Based Sexual Reproductive Health Intervention Safer Choices Programme in Rural KwaZulu‐Natal, South Africa – A Process Evaluation

**DOI:** 10.1002/jia2.70192

**Published:** 2026-10-08

**Authors:** Natsayi Chimbindi, Ursula Ngema, Janet Seeley, Andrew Gibbs, Abigail Harrison, Alun Davies, Stefan Baral, A. Rani Elwy, Maryam Shahmanesh

**Affiliations:** ^1^ Africa Health Research Institute KwaZulu‐Natal South Africa; ^2^ Institute of Global Health University College London London UK; ^3^ University of KwaZulu‐Natal KwaZulu‐Natal South Africa; ^4^ London School of Hygiene and Tropical Medicine London UK; ^5^ Gender and Health Research Unit South African Medical Research Council Pretoria South Africa; ^6^ University of Exeter Exeter UK; ^7^ Brown University Providence Rhode Island USA; ^8^ University of Oxford Oxford UK; ^9^ Johns Hopkins School of Public Health Baltimore Maryland USA; ^10^ Warren Alpert Medical School of Brown University Providence Rhode Island USA; ^11^ Center for Healthcare Optimization and Implementation Research VA Bedford Healthcare System Bedford Massachusetts USA

**Keywords:** adaptation, evaluation, HIV, intervention, learners, sexual reproductive health

## Abstract

**Introduction:**

We co‐adapted and evaluated a multi‐component whole‐school health‐promotion Safer Choices programme to improve sexual and reproductive health (SRH) and uptake of HIV‐prevention among learners in rural uMkhanyakude district, KwaZulu‐Natal, South Africa.

**Methods:**

From January 2023 to June 2025, we used the Framework for Reporting Adaptations and Modifications‐Enhanced (FRAME) to guide and document Safer Choices adaptations. We used participatory qualitative approaches and a process evaluation to assess intervention acceptability and feasibility. Co‐adapting involved a 3‐day workshop with 16 learners aged 15–19 (Grade 9–10), four discussions with school leadership and two focus group discussions (FGDs) with parents/guardians, to understand the context and social validity of the programme. Intervention acceptability was assessed through five in‐depth interviews with teachers/school leadership and three with peer leaders, two workshops with intervention and clinical delivery teams, two FGDs with community members and five with learners. Interactions were audio‐recorded, transcribed and analysed using thematic analysis.

**Results:**

Schools were described as learning spaces providing opportunities for health promotion through health and well‐being lessons. Teachers and parents/guardians noted a need for Safer Choices to provide SRH/HIV services in schools, as most learners were already sexually active. Provision of mobile SRH/HIV services in schools was perceived to be feasible if negotiated with the school and HIV‐status neutral services were provided to minimize stigma. Adaptations to Safer Choices were focused on optimizing intervention delivery or emphasizing social validity of the programme. During implementation of Safer Choices, myths and misconceptions around HIV and pregnancy were addressed. Challenges such as no electricity and large classes made it difficult to deliver Safer Choices. Peer navigators (lay youth‐workers) were engaged to help deliver the intervention.

**Conclusions:**

In a context of high sexual risk and vulnerability, it was feasible to co‐adapt and deliver the Safer Choices programme, which provided accurate and accessible SRH information and services to address adolescent SRH needs of learners in our rural schools. Adaptation challenges included balancing fidelity to intervention with both the “real‐world” context and the practicality of peer navigators supporting intervention delivery. These results suggest that optimizing the impact necessitates appropriately resourcing school‐based interventions to address the challenges of programming in rural settings.

## 1 Introduction

Despite the availability of a wide range of HIV‐prevention and contraception technologies, young people remain at risk of HIV and early pregnancy in South Africa [1, 2, 3, 4, 5]. Although HIV incidence has been declining in the past decade, mostly driven by HIV treatment, incidence remains high among young people aged 15–24 years in South Africa, KwaZulu‐Natal. Incidence declined by 44% in women 15–19, 24% in women 20–24 versus 64% in men 15–19, 68% in men 20–24 for periods 2004–2019 [3, 6], sexually transmitted infections (STIs) prevalence was high among 15‐ to 19‐year‐old (29.2%) with a higher prevalence among females (37.2%, 95% CI: 34.4%–40.1%) than males (17.9%, 95% CI: 15.4%–20.8%) [7] and teenage pregnancy continuing to rise (teenage pregnancy incidence was 9.86 per 100 person‐years in 2019) [7, 8, 9, 10, 11], leading to poor health and well‐being outcomes and socio‐economic livelihood challenges of the adolescents due to school disruptions including drop‐out [12, 13]. In our rural area in northern KwaZulu‐Natal, South Africa, there remains a high unmet sexual health need among young people, including poor uptake of existing sexual and reproductive health (SRH) and HIV prevention and care services in this setting [5, 7, 14, 15, 16, 17].

Challenges exist for young people in accessing SRH and HIV prevention services, particularly in rural areas in South Africa [15, 18], including distance to facilities, inconvenient operating hours, stigma associated with seeking SRH and HIV services, and misconceptions about use of these services [15, 18]. The WHO's Global School Health Initiative has recognized schools as an opportunity to promote and deliver effective health interventions within a sustainable infrastructure [19]. Schools are a critical setting for delivering SRH interventions in South Africa, given school attendance at more than 90% [20], providing an opportunity to reach a large proportion of adolescents with effective programmes [21]. In South Africa, the Departments of Basic Education and Health are jointly implementing the Integrated School Health Programme (ISHP) to provide school health services including health education, health screening and some on‐site health services to all learners in primary and secondary schools [22] and the life skills subject Life Orientation (LO), integrated within the school curriculum to deliver HIV prevention education. The ISHP has challenges reaching learners due to a shortage of resources, while the LO subject was found to be effective in improving HIV‐related knowledge and attitudes but not impacting on behaviour changes among learners [23]. Studies have shown limited behavioural impact of LO on condom use, pregnancy, HIV incidence and STIs due to suboptimal implementation fidelity and quality of delivery due to limited delivery time, high teacher workloads, teacher capacity to teach the subject and unclear referral pathways [24, 25, 26, 27]. Multi‐component programmes that go beyond increasing knowledge to influencing norms, self‐efficacy and service provision might overcome these barriers. Most schools in the uMkhanyakude district, the study site of the current paper, are poorly resourced and no‐fees schools [28] with few targeted HIV/SRH interventions and very little or no school‐based delivery of health services except for screenings done through the ISHP in the district.

The WHO Health Promoting Schools (HPS) framework focuses on the whole‐school approach to improve learners’ health and well‐being through addressing the whole‐school environment, and interventions using the HPS approach have been found effective in improving learners’ health and well‐being outcomes [29]. While existing SRH interventions have shown promise [30, 31], their effectiveness is often context‐dependent, and few efforts have taken place to systematically adapt and evaluate them in rural settings. Adapting whole‐school health‐promotion programmes is important for tailoring interventions to fit the specific needs and context of the schools and communities in which the intervention is delivered to enhance acceptability and feasibility of the intervention [32]. Multi‐component whole‐school health‐promotion programmes to improve SRH and uptake of HIV‐prevention are key and need to be fit for context. The goal of this project was to co‐adapt (a collaborative adaptation process that applies co‐creation principles to either adapt an intervention to a new context and/or adapt the co‐creation process itself) [32] a theoretically derived, evidence‐based multi‐component whole‐school SRH health‐promotion Safer Choices programme for implementation in a rural area of uMkhanyakude district with high HIV prevalence and teenage pregnancy in KwaZulu‐Natal, South Africa. Adapting Safer Choices in rural settings with high SRH unmet needs addresses both the epidemiological need and the context‐specific barriers. Safer Choices is an evidence‐based whole‐school intervention that was developed in the United States, and in trials, it demonstrated effectiveness in improving condom use, reducing unprotected sex and enhancing other psychosocial factors related to sexual health among high school learners [33]. This paper describes our process of co‐adaptation, refining, evaluating and implementing the Safer Choices programme in rural KwaZulu‐Natal, South Africa to ensure feasibility and acceptability of the programme for future widespread implementation.

### 1.1 Safer Choices Programme

Safer Choices represents a school‐based multiple‐component whole‐school sexual health‐promotion educational intervention to reduce sexual risk behaviours and increase protective behaviours in preventing HIV, STIs and pregnancy among high‐school learners [33]. The Safer Choices intervention is based on social cognitive learning theories and the programme's effectiveness is accounted to the intervention's multiple components, its focus on school‐wide change and the influence of the total school environment on student behaviour [33]. The programme targets Grade 9 and 10 (i.e. second‐ and third‐year high school level, respectively) learners and is taught over 2 years (10 lessons for Grade 9 and 11 for Grade 10). The components and activities are shown in Box 1.

**Box 1: Safer Choices multi‐components and activities**

**Table:** 

Component	Activities
School organization	School Health Promotion Council is established and made up of teachers, students, parents and community members. The Council is responsible for organizing and planning the programme activities.
Curriculum and staff development	21 curriculum‐based sessions (Grade 9–10), which use interactive activities to provide information about HIV and sexually transmitted infections (STIs); teach effective condom use, refusal skills and decision‐making skills; and promote positive attitudes and norms about refusing sex and using condoms. The curriculum includes in‐class peer leaders to facilitate selected activities (e.g. leading small‐group role playing) and is implemented by classroom teachers.
Peer resources and school environment	Peer teams/clubs reinforce the curricular messages in the broader school environment through school‐wide activities, events and services designed to alter the normative culture of school.
Parent education	Student‐parent homework activities engage parents in the programme's goals through newsletters, child‐parent discussions on sexuality topics and parent education workshops or speakers. Student‐parent homework activities facilitate communication regarding HIV, other STIs and pregnancy.
School/community linkages	Access to support services such as local information hotlines, clinics and testing services. Students receive homework assignments that require them to find out more about the services available in the community.

## 2 Methods

### 2.1 Study Site

This study was conducted at the Africa Health Research Institute (AHRI), which is a health and demographic surveillance area situated in uMkhanyakude district in KwaZulu‐Natal, South Africa. The district is predominantly rural and poor, with a high burden of HIV, a prevalence of about 30% and high rates of unemployment of about >80% among those aged 18 and above who are out of school [5]. Although most schools are among the poorest quintiles 1–3 [28], school attendance is generally high >75% [5] in the district.

### 2.2 Study Design

We conducted the study between January 2023 and June 2025. We used a combination of participatory workshop approaches to stimulate discussion, suggestions and consensus, with qualitative approaches for deeper exploration of individual and specific group views for co‐adaptation of the Safer Choices programme and a process evaluation to assess intervention acceptability and feasibility across six purposively selected high schools to represent both rural/urban schools in the study area focusing on learners aged 15–19 years in Grade 9–10. We used purposive sampling by grade, class, rural/urban, age, sex, and stakeholder role to recruit learners, stakeholders and community members with diverse perspectives relevant to adapting and evaluating Safer Choices until we reached thematic saturation (when no new data was emerging) for the adaptation/evaluation.

Three processes were involved in this study: (1) co‐adaptation and refining of the Safer Choices programme; (2) intervention delivery of the adapted programme; and (3) evaluation of programme. Figure 1 shows a summary of the adaptation and evaluation process and the approaches used.

**FIGURE 1:**
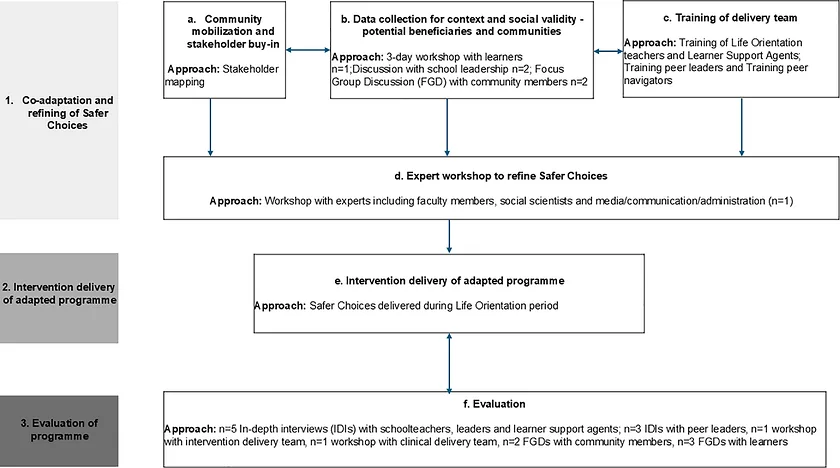
Adaptation and evaluation process and approaches used.

#### 2.2.1 Co‐Adaptation and Refining of Safer Choices

##### 2.2.1.1 Community Mobilization and Stakeholder Buy‐in

The research team and the AHRI Advancement Unit identified potential high schools and stakeholders to work with. We mapped and engaged the stakeholders to get their buy‐in and support; stakeholders included the Department of Education, school‐governing bodies and learner support agents (LSAs). LSAs are professionals allocated to schools to support vulnerable learners and provide counselling and onward referrals when needed.

##### 2.2.1.2 Data Collection for Context and Social Validity—Potential Beneficiaries and Communities

We selected one high‐school serving urban and rural learners and conducted a 3‐day participatory workshop with (*n* = 16) learners in Grade 9–10 (aged 15–19) and held (*n* = 2) discussions with school‐leadership – one with the principal and deputy principal and another with head of department and Life Orientation teacher – and (*n* = 2) focus group discussions (FGDs) with parents/guardians of learners attending the school. These aimed to identify areas to adapt the Safer Choices programme to fit our context, content and delivery of intervention and assess social validity of the programme.

##### 2.2.1.3 Training Intervention Delivery Teams

We trained (*n* = 17) Grade 9–10 Life Orientation teachers and LSAs from six schools focusing on the content and delivery of the curriculum‐based components using participatory approaches such as role plays. We also trained peer leaders (who are learners identified by their peers and teachers to be champions of the programme) within the schools, focusing on understanding the Safer Choices curriculum, peer and schoolwide activities. Last, we trained AHRI peer navigators who are lay‐youth leaders working in communities served by the schools to support schools with programme delivery and referral and linkage to mobile health clinics for learners. During training, we identified areas requiring adaptation/modification and support required. After training, we verbally assessed understanding of content and delivery methods and social validity of the programme.

##### 2.2.1.4 Experts Workshop to Refine Safer Choices

We documented all adaptations and modifications suggested during the interviews, discussions and training sessions and presented these for discussion in a workshop with intervention and evaluation experts who were not involved directly with the study. Experts (*n* = 10) included AHRI senior research scientists (behavioural scientists, social scientists, psychologists, mental health experts), post‐doctoral social science researchers and administration support – media and communication. The adaptations/modifications identified were sent out ahead of the workshop together with a list of definitions of terms of implementation science concepts to generate common understanding. A Robot/Traffic light system adapted from the Safer Choices manual [34] was used to rank modifications/adaptations as follows: Green Light – Adaptations are safe, Yellow Light – Adaptations should be made with caution and Red Light – Adaptations are unsafe and should be avoided. Appendix 1 shows the workshop outline and the list of adaptations/modifications considered.

We did refresher training of the adapted Safer Choices with the delivery teams (teachers, LSAs, peer leaders, peer navigators) before implementation.

#### 2.2.2 Intervention Delivery of Adapted Safer Choices Programme

**Intervention Delivery of Adapted Programme**. Delivery was teacher‐ and peer‐led, supported by peer navigators. Time to deliver the curriculum‐based component in schools was negotiated with the school leadership and LO teachers. Implementation was rolled out simultaneously across five schools during school term three from August and completed in term four in December. Parental components of the programme were sent home with learners, while peer team activities and mapping of community resources were done alongside the curriculum delivery. Regular debriefing meetings were held with the delivery team to assess how and which components were delivered, using a checklist, and identify any modifications which would be documented. Appendix 2 shows templates of the (a) school schedule; (b) checklist used to check completion of delivery; and (c) debriefing form used with the team to ensure fidelity of intervention delivery. The senior research assistant and the researcher, NC, often accompanied the teams to the school to observe and support delivery of intervention. A participatory workshop with the school curriculum delivery team (total *n* = 22) was held in addition to the regular debriefs to understand their experience of delivering the intervention, challenges and opportunities and if the intervention was delivered as intended. All the data were analysed qualitatively and thematically as part of the evaluation data.

#### 2.2.3 Evaluation of Safer Choices Programme

**Evaluation**. We conducted a process evaluation including in‐depth interviews (IDIs) with teachers/school leadership, learner peer‐leaders, and held workshops with peer navigators and the clinical delivery team (these are nurses and clinical research assistants who were providing services at the mobile clinics) and FGDs with community members, to assess acceptability of intervention, and identify facilitators and barriers for reach/delivery, and integration with biomedical services. We conducted (*n* = 5) IDIs with teachers/school leadership, (*n* = 3) peer‐leaders, (*n* = 1) workshop with intervention delivery team, (*n* = 1) workshop with clinical delivery team and (*n* = 2) FGDs with community members, and (*n* = 3) FGDs with learners to assess intervention acceptability.

### 2.3 Data Collection

#### 2.3.1 Qualitative

We used qualitative approaches including FGDs, structured discussions, IDIs and participatory workshops to collect data and adapt the programme (Table 1). These were conducted by two trained social scientist research assistants who were both native isiZulu speakers, the first language of the participants. IDIs took between 30 and 60 min each, while FGDs took 1–2 h. Separate male and female FDGs with learners were held to maximize learner's ability to voice opinions, particularly on sex‐related matters and mixed‐sex group discussions to allow for a broad range of ideas and perspectives. Participatory workshops were conducted with groups ranging from 10 to 22 participants and ranging from 2h to half‐day and up to 3 days as in the case of learner workshops. Data collection tools were developed and piloted internally at AHRI. At the end of IDIs and FGDs, participants were provided with a light snack and an airtime voucher of R50 (∼3USD). All interactions were audio‐recorded, transcribed verbatim and translated from isiZulu to English.

**TABLE 1:** Characteristics of participants by research phase and activity.

Research phase	Research activity	Characteristics	Number of participants
**Adaptation**	Safer Choices 3‐day learner workshop	Learners Grade 9–10 both girls and boys	16
	Structured discussion school management	Principal and deputy principal (*n* = 1), two males	2
	Structured discussion teaching staff	Life Orientation and Head of Department (*n* = 1), two females	2
	Focus group discussion community members	Community members who are parents/guardians of learners (*n* = 1) three males and one female	4
		Community members who are parents/guardians of learners (*n* = 1) females only	5
	Expert workshop	Experts including faculty members, social scientists and media/communication/administration (*n* = 1)	10
**Process evaluation**	In‐depth interviews with delivery team	Life orientation teachers (*n* = 2) 38‐year‐old female; 42‐year‐old female	2
		Learner support agent (*n* = 1) 36‐year‐old female	1
		Head of Department (*n* = 1) one female	1
		Deputy Principal (*n* = 1) one male	1
		Learner peer leader (*n* = 3) 17‐year‐old male, 16‐year‐old female, 17‐year‐old female	3
	Focus group discussion potential beneficiaries – learners and community members	Community members (*n* = 1) three males and one female	4
		Community members (*n* = 1) eight males and two females	10
		Learners (males only) (*n* = 1) eight males	8
		Learners (females only) (*n* = 1) eight females	8
		Learners (mixed group) (*n* = 3) four males and four females in each group	24
	Participatory workshop – delivery team	School curriculum delivery team – peer navigators, research team (*n* = 1), both males and females	22
		Clinical delivery team – clinical research assistants and nurses (*n* = 1), both males and females	16
		**Total participants**	**139**

### 2.4 Data Analysis

We used the Framework for Reporting Adaptations and Modifications to Evidence‐based (FRAME) [32] to guide and document modifications and adaptations to Safer Choices. We used the framework's components to assess modifications identified and the possible impact of these. Qualitative data were analysed by the two researchers and overseen by the senior researcher using thematic analysis [35]. Regular debriefings were held to review and dis/agree on codes. Data were coded separately and compared using a matrix display to identify convergent and divergent perspectives. We identified both inductive and deductive themes and iteratively coded the data; all data and analysis were managed using NVivo 12 software [36]. We used data triangulation by comparing findings across learner workshops, stakeholder interviews, community members discussions and teacher, peers and peer navigators training for adaptation and interviews and discussions with beneficiaries (learners), stakeholders, delivery teams and community members for evaluation. Discrepancies were discussed during debriefing meetings and resolved by team consensus.

### 2.5 Ethics

Ethical approval was granted by the University of KwaZulu‐Natal's Biomedical Research Ethics Committee (BREC) (BREC/00005024/2022), and permission from the AHRI Somkhele Community Advisory Board. We obtained parental waiver for learners who are minors (<18 years); thus, we obtained written informed assent from minors and written informed consent to participate from all the participants >18 years prior to participating in the study.

## 3 Results

We recruited a total of *n* = 139 participants across the six schools and their communities. Table 1 shows characteristics of participants by the research phase and activity.

We present the findings in three parts: (1) context and co‐adaptation of the intervention; (2) intervention delivery; and (3) evaluation of the programme.

### 3.1 Context and Co‐Adaptation of the Intervention – Understanding the School Environment for Intervention Delivery

#### 3.1.1 Stakeholders

We approached the Department of Education district‐level office and engaged them on the list of schools and stakeholders, and they advised further on other stakeholders we needed to engage with and how to access schools. Constant engagement with the stakeholders at the department was key to understanding the school environment, its structures and hierarchy from a governance and leadership viewpoint. The department found the Safer Choices programme to be acceptable and aligned with the department's strategy and goals, especially on tackling learner pregnancy and engaging parents and the community in the well‐being of learners.

#### 3.1.2 School Environment

We integrated the Safer Choices programme within the existing school curriculum LO subject. This was acceptable for the school as Safer Choices content was similar to the LO curriculum, and the LO teacher could deliver the programme during these subject periods:

*“Safer Choices curriculum‐based component can work well with LO teachers. There is a need for people who will assist with delivering because teachers have their workload.”* (School leadership discussion)

Most schools offered two periods of LO per week per class, and we negotiated with the LO teachers and school management to use one for Safer Choices delivery. Most schools were large and overcrowded, typical of quintile 1 to 3 schools, and class sizes ranged from about 30–60 learners per class with 2–4 classes per grade. Teachers usually took learners for more than one subject, and their workload was huge. Although teachers expressed interest in delivering Safer Choices, most of them could not go beyond setting the scene in classes and arranging the LO periods for Safer Choices delivery due to their high workload, and this necessitated collapsing the programme from 21 to 11 sessions and involving peer navigators to support delivery of Safer Choices in class. Only two out of the six schools had an LSA assigned to the school who could assist delivering Safer Choices. LSAs were essential in the delivery of Safer Choices in these schools as the programme dovetailed with their roles.

#### 3.1.3 SRH Needs and Risks

We found that schools are characterized mostly as academic spaces that focus on educational attainment. However, subjects such as LO incorporated health and well‐being aspects, which provided an opportunity for health promotion. Although schools were learning spaces, high sexual risk and vulnerability were reported, including learner pregnancy, alcohol and substance abuse and violence and bullying were reported to be common in some schools, as one learner reported:

*“when you go to the toilet, boys will be there waiting and ask you for money if you want to use the toilet”* (Female, learner workshop).

Teachers were reported to sometimes deliver incorrect fear‐based messaging around contraception to discourage sexual engagement. Such messaging and lack of accurate SRH information is dangerous in a setting with high sexual risk. One learner reported:

*“but sir told us that contraceptive pills can destroy fallopian tubes if you drink them religiously”* (Female, learner workshop)

#### 3.1.4 Refining and Co‐Adapting Safer Choices – Expert Workshop

We identified several necessary adaptations to the Safer Choices programme from the discussions and interviews. The modifications included changes to components/domains including content/concepts, for example “lack of sessions on HIV pre‐exposure prophylaxis (PrEP), bullying and harassment,” delivery, for example “need for summaries or slides for lesson planning,” social validity/appropriateness, for example “teachers not willing to do condom demonstration” and context, for example “need to consider wording of response options such as ‘fight back’ in the context of gender based violence.” Safer Choices programme had a movie as part of the curriculum component which needed to be relatable to context. There was a suggestion to use a local edutainment‐drama that resonated with young people in our context called MTV Shuga [11, 37] (an edutainment drama that featured on national television which aims to educate young people about SRH, HIV prevention, sexual assault and gender‐based violence with the aim to improve knowledge, attitudes and behaviours towards HIV prevention).

Key examples of adaptations identified under each component/domain are described in Table S1 and in Appendix 1. Some of the adaptations suggested were crosscutting between components/domains, for example teachers’ refusal to conduct condom demonstrations in class was influenced by it not being seen as appropriate and a delivery mode they were not confident they could do. Teachers reported that they are often seen as parents or respected guardians within the school environment and therefore, they were uncomfortable to do condom demonstration in front of their “children” despite it being a component in the LO curriculum. Changes were mostly adaptations to optimize intervention delivery, or to emphasize social validity – appropriateness and acceptability of the programme and the context in which the programme would be delivered. We did not adapt modifications which would affect the core components of the intervention, or the study aims, for example removing condom demonstration or practise lesson or using visual aids to show how STIs look like to support linkage to care (Table S1 and Appendix 1). Below is a summary of not/adapted modifications (Table 2).

**TABLE 2:** Summary of adaptations/modifications adopted and declined.

Adaptation	Outcome/decision
Outdated HIV and STI estimates from the United States of America, to be replaced with current South Africa estimates	Adapted
Condom use demonstration to be delivered by peer navigator instead of teachers	Adapted
Take out learner condom demonstration practise using models – fear of push back from parents	Not adapted – a core component of Safer Choices programme, learners free not to participate.
Add HIV pre‐exposure prophylaxis (PrEP) awareness and uptake to the curriculum	Adapted
Use a local edutainment‐drama that relates to South Africa context such as the MTV Shuga instead of the one in Safer Choices	Adapted
Show both Safer Choices movie and MTV Shuga – so that young people can understand that HIV acquisition does not know any racial/ethnicity lines.	Not adapted – learner time implications.
Parental component of Safer Choices which includes Homework/Quizzes and activities with parents/guardians could be problematic for learners from child‐headed households and sexual reproductive health (SRH) conversations with older generation (grandparents).	Adapted – programme allows for discussions with an older sibling, friend or person they can trust and are open to.
Add visuals/pictures to show how sexually transmitted infections (STIs) look to make the messaging clearer – no pictures are included.	Not adapted – difficult to find the appropriate pictures for the disease stage and using extreme cases may result in missing those with minimal symptoms.

### 3.2 Intervention Delivery – Implementation of Safer Choices

#### 3.2.1 Opportunities

The research team met regularly with the education district office and school leadership to keep them updated on the progress of the study, which established important relationships with key stakeholders. This enabled continual buy‐in and smooth flow of the study and engagement with the programme as part of the school council component. During the workshop held with the intervention delivery team, we found that Safer Choices delivery provided an opportunity to correct SRH misinformation and demystify myths and misconceptions around HIV acquisition, transmission and prevention as well as STI management and SRH issues. Some of the misinformation identified and corrected during delivery included: “*circumcised boys can still get HIV*,” “*condom use with a virgin is important*” *and* “*STIs are treatable*.” The delivery team used the opportunity to encourage learners to access mobile clinics and provided pamphlets with the services and times these mobile clinics would be near their schools. Participatory approaches such as role plays and condom demonstrations were acceptable approaches in these schools where teaching is often didactic. Participatory approaches made learning fun and engaging. The MTV Shuga edutainment drama was captivating for the learners and provided learners with the opportunity to discuss difficult topics such as pregnancy termination and relationships. Similarly, condom demonstration was one of the most eye‐opening activities for the learners, with some learners learning about the female condom for the first time.

*“Learners were helped a lot when talking about teenage pregnancy and abortion – the females opened wide their eyes – the abortion part helped the girls and they spoke to each other after the drama. The boys saw that what the doctor [who did the backdoor abortion] did was wrong. The part on the gay youth triggered discussion [it] showed learners do not understand and were not exposed to it. Drama taught them to choose good friends not sugar daddies or cheeseboys (slang for someone who grew up in the city and is unfamiliar with rural life) – it will destroy their future”* (Peer navigator, workshop).

We used the opportunity of the intervention delivery team workshop to assess fidelity of delivery of the intervention and found that all lessons/components were delivered in all schools. Schools that had greater teacher involvement (four out of five) (in controlling the class, setting the tone, supporting the programme, ensuring the learners did not miss on other school benefits, e.g. school feeding time, etc.) and that had LSAs (two out of five) were more organized and were easier to deliver the intervention than those without.

#### 3.2.2 Challenges

One of the main challenges with delivery of the programme was the huge class sizes and timing of delivery. Large class sizes made it difficult for learners to participate effectively. However, the presence of teachers and the support of peer navigators and compressing of the curriculum ensured that all the sessions were delivered with fidelity. It was particularly difficult to access schools and learners during examination time. This resulted in adapting the implementation strategy from delivery during LO periods only to block delivery over 2–3 days per school. This was acceptable in most schools and was feasible because a large delivery team was available at any given point to support delivery per school. Last, some of the challenges included lack of electricity in some schools for viewing the edutainment drama MTV Shuga due to bad weather or schools lacking money to buy electricity units, which was solved by the research team buying electricity units, indicating some of the programming and structural challenges faced in these poor‐resourced schools. The workshop with the clinical team raised clinical recommendations that if the mobile clinics could provide broader services like the public health clinics, for example antenatal care, implants insertion and removal, and circumcision, which they refer learners to government clinics.

### 3.3 Evaluation

Safer Choices was found to be relevant for learners; the content was appropriate and relatable and provided learners with information and skills on how to protect themselves from unplanned pregnancy, HIV and STIs. Teachers, learners and parents welcomed a school‐based SRH programme that included parents, particularly if it tackled wider well‐being and access to biomedical services. There was also a suggestion to include enrichment and self‐development initiatives alongside Safer Choices, such as, for example driving lessons, choirs, sports and drama for learners in these rural schools, where these were lacking.

Provision of biomedical services and mobile clinic services within the school environment through Safer Choices was seen to be acceptable and feasible if it were HIV‐status neutral and provided general services to be less stigmatizing. School leadership and teachers also indicated that location and time of services provision would need to be negotiated with the school leadership and learners for delivery to be successful.

This was further supported by parents/community members that accessing care was already challenging, and providing only SRH/HIV services for learners reduces acceptability:

*“Offering SRH services only at mobile clinics will create stigma. Most people do not like to use clinics that are in their areas, some learners may prefer to use clinics far from the school; but if the mobile clinics offer general health services and not just SRH, it will help learners’ attend clinics.”* (Female, Community member, FGD).

Teachers and parents/guardians described a need for Safer Choices to provide SRH/HIV services in schools, as most learners were already sexually active and girls were becoming pregnant at an early age:

*“Condoms should be supplied to learners at school because they are already engaging in sexual activities. The condoms will not encourage learners to have sex because they are already having sex. Giving out condoms in schools is better than seeing a 13‐year‐old pregnant at school.”* (Male, Community member, FGD).

The clinical delivery team reported Safer Choices provided critical SRH knowledge for learners, including those already sexually active and emphasized the need for parental involvement in the programme to support adolescent SRH understanding. Last, they also highlighted the need for shorter clinical assessment tools for learners and improved internet network connections to reduce waiting time at the mobile clinic visits.

## 4 Discussion

This study found that the multi‐component health‐promotion SRH Safer Choices programme was acceptable and feasible to deliver in this rural setting of South Africa. This can be attributed to the comprehensive approach engaging multiple groups in co‐adapting and evaluating the programme, which included using participatory approaches and continuous stakeholder engagement to enhance context and social validity. Co‐adaptation of programmes improves the fit between the intervention and context for delivery of relevant, culturally acceptable and appropriate interventions [32, 38].

The co‐adaption process enabled us to understand the school and wider context, which meant that as we co‐adapted Safer Choices we could maintain fidelity, while ensuring it did not “clash” with existing processes/systems. Schools were focused on learning and academic achievements, with high teachers’ workload due to large classes, with few or no enrichment programmes for learners. Integrating Safer Choices within the existing school LO curriculum, compressing the curriculum component and including peer navigators to support delivery were some of the adaptations made which ensured fidelity of delivery, while responding to the context of over‐stretched schooling systems. Safer Choices content and participatory approaches such as role plays, condom demonstrations and edutainment drama [11, 37] were found to be relevant and engaging methods of learning which were different from the usual didactic teaching approaches.

An important aspect of this study was the iterative learning in the co‐adaptation process, particularly the evaluation process conducted alongside intervention delivery. Safer Choices has been evaluated in high‐income countries and found to be effective in increasing condom use and delaying sexual debut [33, 39] but has not been adapted or evaluated for use in rural low‐ and middle‐income countries. Adapting the intervention to rural KwaZulu‐Natal, we found that suggested programme modifications/adaptations were mainly to optimize intervention delivery or emphasize social validity for the context, rather than content modification. Understanding meaning and language was key, for example “fight back” as a way of exerting assertiveness was translated by participants to mean physical fighting during the co‐adaptation process. This necessitated the need to give contextual meaning and include examples of how this would look like in our setting. Similar to what has been found with delivery of some components of the LO [40], teachers across all schools were not comfortable conducting condom demonstration in the classroom, a core component of the intervention. This demonstration was adapted to be delivered by the peer navigators, as this was more appropriate for this setting. This indicates some of the embedded cultural beliefs and norms within these rural communities which may affect delivery and uptake of SRH services by young people. Training of teachers, key stakeholders and communities may be crucial to bridge this gap and improve access and uptake of SRH and HIV prevention services.

The necessary adaptations of Safer Choices show how the tension between fidelity and adaptation for context can be navigated while maintaining the core intervention ingredients/components/mechanisms and the adaptable periphery for context and social validity. For example, shortening the curriculum and introducing peer navigators maintained “function” of skills practice and delivery of Safer Choices while addressing the realities of our rural schools’ teacher workload and time constraints.

The co‐adaptation process also identified some limitations, including challenges of balancing fidelity to the intervention with the “real world” context and balancing ensuring fidelity and practicality. The structure of school timetables and their focus on delivering academic progress was a major challenge. It was particularly difficult to access schools and learners during examination time, which led to a deviation from delivering the intervention alongside LO lessons once or twice a week, to a block delivery over 2–3 days per school and the compressed curriculum, which might have affected the learners’ ability to reflect on concepts [41]. Structural challenges of electricity shortages are not only particular to our rural schools but also to most poor‐resourced schools in South Africa, but this limited learners’ ability to watch drama components. Finally, the shortened curriculum of 11 sessions (from 21) was a compromise shaped by the constraints of teaching time. Although school attendance is generally good in our setting, learners absent on delivery days missed on the intervention, which is a limitation of school‐based programmes failing learners who drop out or are out‐of‐school. Despite these limitations, the study has notable strengths. The peer navigator support helped overcome high teacher workload challenges and ensured delivery of intervention across all schools, and they were able to address existing SRH and HIV misconceptions during delivery. Engagements with the Department of Education enabled access to schools and successful programme delivery that benefit learners. In addition to the work presented in this paper, we conducted a learner pre‐post intervention survey to assess the effect of the intervention on reported change in SRH behaviours in learners. We also collected data at the clinical level to assess linkage to care to the mobile health services provided. These data will be presented separately in the outcomes paper and will provide evidence on whether the programme produces meaningful SRH improvements and motivation for continuity or expanded investment.

Although Safer Choices programme was developed in the United States, adapting the intervention to a South African rural setting contributes to the global evidence base on school‐based HIV prevention for adolescents. Similar to the adapted Stepping Stones programme [42], our findings show that multi‐level approaches which address knowledge, behaviour change and access to services are acceptable and feasible to deliver and generalizable across high‐ and low‐income settings. Compared to the DREAMS programme, which focused only on adolescent girls and young women [14], our findings show that the adapted programme that includes both male and female learners and peer navigator support was acceptable and has potential to reach many learners with services.

## 5 Conclusions

In conclusion, in a context of high sexual risk and vulnerability, it was feasible and acceptable to co‐adapt and deliver a multi‐component whole‐school SRH programme which provided accurate and accessible SRH information and services to address adolescent and sexual health needs of learners in our rural schools. Taken together, these findings highlight the importance of adapting and evaluating evidence‐based programmes to meet the unique needs of rural schools. However, school‐based interventions need to be resourced appropriately to address the challenges of programming in rural settings.

## Author Contributions

NC, MS, UN, JS, AG, AD, SB, ARE, AH, NC and MS conceptualized the study, and NC, MS, JS, AG and UN developed the study tools and performed the research. NC, MS, JS, AG, AH and AD designed the research study. NC and UN supported by MS, JS, AG, AD and SB, ARE conducted the analysis and NC wrote the first and final draft of the paper with input from MS, JS, AG, AD, SB and ARE. All the authors critically reviewed the manuscript. All authors have approved the final draft of the paper.

## Funding

Africa Health Research Institute is supported by core funding from the Wellcome Trust (Core grant number 082384/Z/07/Z). MS is an NIHR Research Professor (NIHR 301634). NC is supported by a Wellcome Trust Early Career Fellowship (grant number 224309/Z/21/Z). ARE is funded by a U.S. Veterans Affairs Research Career Scientist Award, RCS23‐081. For the purpose of open access, the author has applied a CC BY public copyright licence to any Author Accepted Manuscript version arising from this submission. The funders have played no role in the study design, writing of the manuscript and in the decision to submit the manuscript for publication.

## Conflicts of Interest

The authors declare no conflicts of interest.

## Supporting information




**Appendix S1**: Workshop to adapt and refine the Safer Choices program with experts' group.

**Appendix S2**: a. School schedule, b. checklist used to check completion of delivery and c. debriefing form.

**Table S1**: Adaptations/Modifications and action adopted.

## Data Availability

The data that support the findings of this study are available on request from the corresponding author. The data are not publicly available due to privacy or ethical restrictions.

## References

[1] UNAIDS , Young People and HIV (2021).

[2] Human Sciences Research Council (HSRC) , The Fifth South African National HIV Prevalence, Incidence, Behaviour and Communication Survey (South African Health Services Research Council, 2017).

[3] Human Sciences Research Council (HSRC), Turning the Tide – SA HIV Survey Shows Encouraging Trends in Prevalence, Prevention and Treatment (HSRC, 2024), https://hsrc.ac.za/press‐releases/sabssm/turning‐the‐tide‐sa‐hiv‐survey‐shows‐encouraging‐trends‐in‐prevalence‐prevention‐and‐treatment/#:~:text=The%20survey%20found%20that%20HIV, at%2081.4%25%20for%20all%20ages.

[4] K. Baisley , N. Chimbindi , N. Mthiyane , et al., “High HIV Incidence and Low Uptake of HIV Prevention Services: The Context of Risk for Young Male Adults Prior to DREAMS in Rural KwaZulu‐Natal, South Africa,” PLoS ONE 13, no. 12 (2018): e0208689, 10.1371/journal.pone.0208689. PMC6306176 30586376

[5] N. Chimbindi , N. Mthiyane , I. Birdthistle , et al., “Persistently High Incidence of HIV and Poor Service Uptake in Adolescent Girls and Young Women in Rural KwaZulu‐Natal, South Africa Prior to DREAMS,” PLoS ONE 13, no. 10 (2018): e0203193, 10.1371/journal.pone.0203193. PMC6191091 30325932

[6] A. Akullian , A. Vandormael , and J. C. Miller , “Large Age Shifts in HIV‐1 Incidence Patterns in KwaZulu‐Natal, South Africa,” Proceedings of the National Academy of Sciences of the United States of America 118, no. 28 (2021): e2013164118, 10.1073/pnas.2013164118. PMC8285891 34244424

[7] J. Busang , N. Ngoma , T. Zuma , et al., “Person‐Centred HIV Care and Prevention for Youth in Rural South Africa: Preliminary Implementation Findings From Thetha Nami ngithethe nawe Stepped‐Wedge Trial of Peer‐Navigator Mobilization Into Mobile Sexual Health Services,” Journal of the International AIDS Society 28, no. Suppl 5 (2025): e70032, 10.1002/jia2.70032. PMC12505191 41059644

[8] S. C. Francis , T. N. Mthiyane , K. Baisley , et al., “Prevalence of Sexually Transmitted Infections Among Young People in South Africa: A Nested Survey in a Health and Demographic Surveillance Site,” PLoS Medicine 15, no. 2 (2018): e1002512, 10.1371/journal.pmed.1002512. PMC5828358 29485985

[9] J. Jarolimova , J. Yan , S. Govere , et al., “Sexually Transmitted Infection Testing Integrated With HIV Prevention and Contraceptive Services in Hair Salons in Urban South Africa,” Journal of Acquired Immune Deficiency Syndromes 99, no. 4 (2025): 359–367, 10.1097/QAI.0000000000003677. PMC12187152 40192662

[10] G. McHunu , K. Peltzer , B. Tutshana , and L. Seutlwadi , “Adolescent Pregnancy and Associated Factors in South African Youth,” African Health Sciences 12, no. 4 (2012): 426–434. 10.4314/ahs.v12i4.5 PMC3598281 23515418

[11] N. Chimbindi , N. Mthiyane , G. Chidumwa , et al., “Evaluating Use of Mass‐Media Communication Intervention ‘MTV‐Shuga’ on Increased Awareness and Demand for HIV and Sexual Health Services by Adolescent Girls and Young Women in South Africa: An Observational Study,” BMJ Open 13, no. 5 (2023): e062804, 10.1136/bmjopen-2022-062804. PMC10201230 37208144

[12] J. Kelly , C. Coakley , J. Jochim , et al., “Supporting Pregnant and Mothering Adolescents in Their Schooling: Insights for Policy and Practice,” International Journal of Educational Development 117 (2025): 103367, 10.1016/j.ijedudev.2025.103367. PMC13293503 42358849

[13] J. Jochim , L. D. Cluver , and F. Meinck , “Learner Pregnancy in South Africa's Eastern Cape: The Factors Affecting Adolescent Girls' School Withdrawal During Pregnancy,” International Journal of Educational Development 87 (2021): 102484, 10.1016/j.ijedudev.2021.102484.

[14] A. Gourlay , I. Birdthistle , N. T. Mthiyane , et al., “Awareness and Uptake of Layered HIV Prevention Programming for Young Women: Analysis of Population‐Based Surveys in Three DREAMS Settings in Kenya and South Africa,” BMC Public Health [Electronic Resource] 19, no. 1 (2019): 1417, 10.1186/s12889-019-7766-1. PMC6824290 31666043

[15] T. Zuma , J. Seeley , S. Mdluli , et al., “Young People's Experiences of Sexual and Reproductive Health Interventions in Rural KwaZulu‐Natal, South Africa,” International Journal of Adolescence and Youth 25, no. 1 (2020): 1058–1075, 10.1080/02673843.2020.1831558. PMC8224946 34177039

[16] O. Adeagbo , C. Herbst , A. Blandford , et al., “Exploring People's Candidacy for Mobile Health–Supported HIV Testing and Care Services in Rural KwaZulu‐Natal, South Africa: Qualitative Study,” Journal of Medical Internet Research 21, no. 11 (2019): e15681, 10.2196/15681. PMC6887816 31738174

[17] K. Baisley , J. Seeley , M. Siedner , et al., “Findings From Home‐Based HIV Testing and Facilitated Linkage After Scale‐up of Test and Treat in Rural South Africa: Young People Still Missing,” HIV Medicine 20, no. 10 (2019): 704–708, 10.1111/hiv.12787. PMC6788950 31454139

[18] L. Nyblade , J. W. Ndirangu , I. S. Speizer , et al., “Stigma in the Health Clinic and Implications for PrEP Access and Use by Adolescent Girls and Young Women: Conflicting Perspectives in South Africa,” BMC Public Health [Electronic Resource] 22, no. 1 (2022): 1916, 10.1186/s12889-022-14236-z. PMC9563466 36242000

[19] WHO , Global School Health Initiative (2021), https://www.who.int/school_youth_health/gshi/en/.

[20] K. Hall , Children's Access to Education (University of Cape Town, 2015).

[21] D. A. Ross , M. L. Plummer , P. Montgomery , et al., “World Health Organization Recommends Comprehensive School Health Services and Provides a Menu of Interventions,” Journal of Adolescent Health 69, no. 2 (2021): 195–196. 10.1016/j.jadohealth.2021.04.036 PMC8288030 34134935

[22] Education DoB , Integrated School Health Policy (Department of Basic Education, 2019), https://www.education.gov.za/Programmes/HealthPromotion/IntegratedSchoolHealthProgramme.aspx.

[23] R. Magnani , K. Macintyre , A. M. Karim , et al., “The Impact of Life Skills Education on Adolescent Sexual Risk Behaviors in KwaZulu‐Natal, South Africa,” Journal of Adolescent Health 36, no. 4 (2005): 289–304, 10.1016/j.jadohealth.2004.02.025. 15780784

[24] A. Jacobs , “Life Orientation as Experienced by Learners: A Qualitative Study in North‐West Province,” South African Journal of Education 31, no. 2 (2011): 212–223, 10.15700/saje.v31n2a481.

[25] I. S. Speizer , M. Mandal , K. Xiong , N. Makina , A. Hattori , and D. Durno , “Impact Evaluation of Scripted Lesson Plans for HIV‐Related Content in a Life Orientation Curriculum: Results From Two Provinces in South Africa,” BMC Public Health [Electronic Resource] 20, no. 1 (2020): 1542, 10.1186/s12889-020-09640-2. PMC7556937 33054742

[26] I. S. Speizer , K. Xiong , M. Mandal , N. Makina‐Zimalirana , A. Hattori , and D. Durno , “HIV‐Related Knowledge, Attitudes, and Behaviors Among Grade 10 Girls and Boys in Mpumalanga and KwaZulu‐Natal: Cross‐Sectional Results,” Open AIDS Journal 14 (2020): 75–83, 10.2174/1874613602014010075. PMC7758912 33362883

[27] E. Prinsloo , “Implementation of Life Orientation Programmes in the New Curriculum in South African Schools: Perceptions of Principals and Life Orientation Teachers',” South African Journal of Education 27, no. 1 (2007): 155–170.

[28] Department of Basic Education , Government Bridges Inequality Gap Through No Fee School Policy (2025), https://www.education.gov.za/ArchivedDocuments/ArchivedArticles/Governmentbridgesinequalitygap.aspx.

[29] R. Langford , C. Bonell , H. Jones , et al., “The World Health Organization's Health Promoting Schools Framework: A Cochrane Systematic Review and Meta‐Analysis,” BMC Public Health [Electronic Resource] 15, no. 1 (2015): 130, 10.1186/s12889-015-1360-y. PMC4339015 25886385

[30] M. Simuyaba , B. Hensen , M. Phiri , et al., “Engaging Young People in the Design of a Sexual Reproductive Health Intervention: Lessons Learnt From the Yathu Yathu (“For us, by us”) Formative Study in Zambia,” BMC Health Services Research 21, no. 1 (2021): 753, 10.1186/s12913-021-06696-7. PMC8320161 34325696

[31] G. Oberth , T. Chinhengo , T. Katsande , et al., “Effectiveness of the Sista2Sista Programme in Improving HIV and Other Sexual and Reproductive Health Outcomes Among Vulnerable Adolescent Girls and Young Women in Zimbabwe,” African Journal of AIDS Research 20, no. 2 (2021): 158–164, 10.2989/16085906.2021.1918733. 33998958

[32] S. Wiltsey Stirman , A. A. Baumann , and C. J. Miller , “The FRAME: An Expanded Framework for Reporting Adaptations and Modifications to Evidence‐Based Interventions,” Implementation Science 14, no. 1 (2019): 58, 10.1186/s13012-019-0898-y. PMC6554895 31171014

[33] D. Kirby , E. Baumler , K. Coyle , et al., “The “Safer Choices” Intervention: Its Impact on the Sexual Behaviors of Different Subgroups of High School Students,” Journal of Adolescent Health 35, no. 6 (2004): 442–452, 10.1016/S1054-139X(04)00071-0. 15581523

[34] ETR and CDC Division of Reproductive Health; R. Firpo‐Triplett and T. R. Fuller , General Adaptation Guidance: An Evidence‐Based Program a Guide to Adapting Evidence‐Based Sexual Health Curricula (2012).

[35] V. Braun and V. Clarke , “Using Thematic Analysis in Psychology,” Qualitative Research in Psychology 3, no. 2 (2006): 77–101, 10.1191/1478088706qp063oa.

[36] QSR International Pty Ltd , NVIVO Version 11 (2018).

[37] N. Kyegombe , T. Zuma , S. Hlongwane , et al., “A Qualitative Exploration of the Salience of MTV‐Shuga, an Edutainment Programme, and Adolescents' Engagement With Sexual and Reproductive Health Information in Rural KwaZulu‐Natal, South Africa,” Sexual and Reproductive Health Matters 30, no. 1 (2022): 2083809, 10.1080/26410397.2022.2083809. PMC9448405 35929964

[38] J. de Boer , G. R. Longworth , L. R. Delfmann , et al., “Exploring Co‐Adaptation for Public Health Interventions: Insights From a Rapid Review and Interviews,” BMC Public Health [Electronic Resource] 25, no. 1 (2025): 614. 10.1186/s12889-025-21544-7 PMC11827302 39953479

[39] K. Coyle , K. Basen‐Engquist , D. Kirby , et al., “Safer Choices: Reducing Teen Pregnancy, HIV, and STDs,” Public Health Reports 116, no. Suppl 1 (2001): 82–93, 10.1093/phr/116.S1.82. PMC1913682 11889277

[40] J. Chaskalson , “Chapter 21: Comprehensive Sexuality Education (CSE),” in Basic Education Rights Handbook (2022).

[41] R. Jewkes , S. Willan , L. Heise , et al., “Elements of the Design and Implementation of Interventions to Prevent Violence Against Women and Girls Associated With Success: Reflections From the What Works to Prevent Violence Against Women and Girls?” Global Programme 18, no. 22 (2021): 12129. 10.3390/ijerph182212129 PMC8621962 34831885

[42] A. Gibbs , L. Washington , N. Abdelatif , et al., “Stepping Stones and Creating Futures Intervention to Prevent Intimate Partner Violence Among Young People: Cluster Randomized Controlled Trial,” Journal of Adolescent Health 66, no. 3 (2020): 323–335, 10.1016/j.jadohealth.2019.10.004. 31784410

