# Supplementary material for: Adaptation and Evaluation of a Multi‐Component School‐Based Sexual Reproductive Health Intervention Safer Choices Programme in Rural KwaZulu‐Natal, South Africa – A Process Evaluation
Source: J Int AIDS Soc. 2026 Oct 8;29(Suppl 5):e70192. doi: 10.1002/jia2.70192 (PMC13650518; doi:10.1002/jia2.70192)
Supplement: Supplementary file 1 [file JIA2-29-e70192-s001.docx]

**Appendix 1 – Experts’ group workshop guide and the list of adaptations/modifications**

| 1. **What needs to be adapted?** | 2. **Reasons for adapting/not adapting?** | | | | 3. **Use the traffic light to decide whether to accept/not the change?**   **Robot**  **Green – Go**  **Yellow – approach with caution**  **Red – do not proceed** | 4. **Comments/suggestions for how to adapt**   ***(optional)*** |
| --- | --- | --- | --- | --- | --- | --- |
|  | **Does this change affect the fidelity of the program?**  Think: delivery of program,  Consistency/  Dose of delivery of program | **Does this change affect the core components of the program?**  Think: content of the program,  Structure of the program | **Does this change ‘fit’ with the culture of this community?**  Think:  Is it Relevant,  Acceptable,  Appropriate | **Are there enough resources to effect/not this change?**  Think: Money, Time, Human resources |  |  |
| 1 |  |  |  |  |  |  |
| 2 |  |  |  |  |  |  |
| 3 |  |  |  |  |  |  |
| 4 |  |  |  |  |  |  |
| 5 |  |  |  |  |  |  |
| 6 |  |  |  |  |  |  |
| 7 |  |  |  |  |  |  |
| 8 |  |  |  |  |  |  |
| 9 |  |  |  |  |  |  |
| 10 |  |  |  |  |  |  |
| 11 |  |  |  |  |  |  |

**Use these Adaptations/modifications to fill in table 1 column 1 “What needs to be adapted?”** **Note Classes/Activities can be provided as appendices if needed*

1. Currently Safer Choices program uses outdated HIV and STI estimates from USA (Page 57)*, suggest we use current South Africa estimates?
2. The topic on Refusal skills to have sex with partner (class 6, level 2)* doesn’t include bullying and harassment topics, can they be added/incorporated when delivering this class?
3. Refusal skills (class 6, level 2)* lesson has "Fight Back" as a last resort/option to get yourself out of a situation with your partner (when you do not want to have sex). We suggest maybe replacing with "Run, Remove yourself from situation, or Call someone" as replacement options because the situation can easily get violent
4. For the activity on condom use demonstration (practical demonstration using models) that the teachers are meant to deliver, can it be delivered by someone eg a peer navigator instead? Teachers are too shy to deliver the lesson.
5. Level 1 class 10, Activity 2* which has aspects of learners actually practising condom demonstration in class (using models) to be taken out as there is fear of push back from parents who might feel by teaching learners how to put on condoms we are encouraging learners to have sex.
6. Can we add PrEP awareness and uptake in Safer Choices curriculum?
7. Can we add include enrichment and self-development initiatives alongside Safer Choices such as eg driving lessons, choirs, sports and drama?
8. The Safer Choices program has a DVD/movie component. The movie is called Bloodlines (I can share this if needed) and features mostly white teenagers/young people narrating (no sub-titles) how they acquired HIV. There was a suggestion to use an edu-drama similar to Bloodlines but that relates to SA context such as the MTV Shuga (an edutainment drama that featured on national TV – please check YouTube for snippets - MTV Shuga DownSouth) episodes on person living with HIV.
9. Another suggestion was to show both Bloodlines and MTV Shuga – so that young people can understand that HIV acquisition does not know any racial/ethnicity lines.
10. The Parental component of Safer Choices which includes Homeworks/Quizzes and activities with parents/guardians to promote parental communication could be problematic for learners that are from child-headed households. Also, older generation households – as it might be difficult to start SRH conversations with older generation (grandparents). How should we handle these cases? Note: The current Safer Choices program allows for discussions with an older sibling, friend or person they can trust and are open to.
11. For the STI lesson, can we add visuals/pictures to show how STIs look like to make the messaging clearer? Currently, no pictures are included but rather use of posters to describe transmission, diagnosis and treatment and class participatory activities to show how it is spread.
