# Supplementary material for: Adaptation and Evaluation of a Multi‐Component School‐Based Sexual Reproductive Health Intervention Safer Choices Programme in Rural KwaZulu‐Natal, South Africa – A Process Evaluation
Source: J Int AIDS Soc. 2026 Oct 8;29(Suppl 5):e70192. doi: 10.1002/jia2.70192 (PMC13650518; doi:10.1002/jia2.70192)
Supplement: Supplementary file 2 [file JIA2-29-e70192-s002.docx]

**Supplementary table 1: Adaptations/Modifications and action adopted**

| **Research activity** | **Components/Domain** | **Adaptation** | **Outcome/Decision** |
| --- | --- | --- | --- |
| **Expert workshop** | Content | Current Safer Choices programme uses outdated HIV and STI estimates from United States of America (USA), we suggest we use current South Africa estimates | Green - adapted |
|  | Content | The topic on Refusal skills to have sex with partner does not include bullying and harassment topics, can they be added/incorporated when delivering this class? | Yellow - not adapted but incorporated in Safer Choices discussions |
|  | Context | Refusal skills lesson has "Fight Back" as a last resort/option to get yourself out of a situation with your partner (when you do not want to have sex). We suggest maybe replacing with "Run, or remove yourself from situation, or call someone" as replacement options because the situation can easily get violent. | Yellow - not adapted but incorporated in Safer Choices discussions emphasizing on assertiveness and always having a plan (eg going to the bathroom to make a call, having spare keys, having money to be able to catch a lift back home, have a friend/someone always know where they are and who they are with) on who to call or reach out to when a situation gets out of hand. |
|  | Social validity/Delivery | For the activity on condom use demonstration (practical demonstration using models) that the teachers are meant to deliver, can it be delivered by someone eg a peer navigator instead? Teachers are too shy to deliver the lesson. | Green - adapted. Peer navigators who were supporting delivery of Safer Choices with teachers within the schools, were trained on how to do male and female condom demonstrations for young people as part of their wider safe spaces activities with young people in the communities they work in. |
|  | Social validity | One of the class/activities which has aspects of learners practising condom demonstration in class (using models) to be taken out as there is fear of push back from parents who might feel by teaching learners how to put on condoms we are encouraging learners to have sex. | Red - not adapted. This was considered a core component of the Safer Choices programme and removing it would interfere with impact on outcomes. However, learners would be encouraged to practice in pairs with the help of the peer navigator but those who are not comfortable to do it will not be forced. |
|  | Content | Can we add PrEP awareness and uptake in Safer Choices curriculum? | Green - adapted with emphasis that the prevention method (PrEP) does not prevent against pregnancy and STIs – condoms prevent against all three. |
|  | Context | Can we add/include enrichment and self-development initiatives alongside Safer Choices such as eg driving lessons, choirs, sports and drama? | Yellow - adapted with caution and looked for opportunities to support the enrichment activities through the school council and peer teams by mobilizing resources. We will also assist consolidate existing opportunities with an SRH focus and link with the community linkage component of Safer Choices where possible. |
|  | Context | The Safer Choices programme has a movie component. The movie is called Bloodlines and features mostly white teenagers/young people narrating (with no sub-titles) how they acquired HIV and how that changed their lives. There was a suggestion to use an edutainment-drama similar to Bloodlines but that relates to SA context such as the MTV Shuga (an edutainment drama that featured on national TV - MTV Shuga DownSouth) episodes on person living with HIV.  MTV Shuga uses drama to educate young people about sexual and reproductive health, including HIV prevention, sexual assault, and gender-based violence with the aim to improve knowledge, attitudes, and behaviours, towards HIV prevention. | Green - adapted. We will use the edutainment drama MTV Shuga Down South which – has been aired on national TV; and there has been community-viewings done by AHRI as part of the evaluation of the drama. The findings from the evaluation shows that the drama was relatable and appropriate for the young people in our setting. |
|  | Context/Delivery | Suggestion to show both Bloodlines and MTV Shuga – so that young people can understand that HIV acquisition does not know any racial/ethnicity lines. | Red - not adapted due to resolution on the earlier suggestion. Further, showing both movies had time implications for learners. |
|  | Context | The Parental component of Safer Choices which includes Homework/Quizzes and activities with parents/guardians to promote parental communication could be problematic for learners that are from child-headed households. Also, older generation households – as it might be difficult to start sexual reproductive health (SRH) conversations with older generation (grandparents). How should we handle these cases? Note: The current Safer Choices programme allows for discussions with an older sibling, friend or person they can trust and are open to. | Green - adapted, with emphasis that Safer Choices programme allowed for leaners to find any other adult they felt comfortable with doing the parental/guardian component with. |
|  | Content/delivery | For the STI lesson, can we add visuals/pictures to show how STIs look to make the messaging clearer? Currently, no pictures are included but rather use of posters to describe transmission, diagnosis and treatment and class participatory activities to show how it is spread. | Red - not adapted because it may be difficult to find the appropriate pictures for the disease stage and using extreme cases may result in missing those with minimal symptoms. We agreed to emphasize that learners go to the clinic if they have any symptom/s they are unsure of. |
| **Research team** |  |  |  |
|  | Delivery | Request to make summary slides according to lessons plans to minimize work for teachers. | Adapted - research team prepared summaries ahead of sessions |
|  | Context | The movie component must include parties and alcohol as that is where and when most learners in the area are at risk. | Adapted - MTV Shuga Down South episode selected included these and other risky situations young people find themselves in |
|  | Social validity/delivery | Parents education material must be in isiZulu for most parents to be able to work on the material with learners. | Adapted - all materials were translated |
|  | Context | The English language used in the Bloodlines movie will be a barrier for learners to understand therefore, episodes from MTV Shuga will be better as they are easy to follow by watching the acting. | Adapted - as discussed in the expert’s workshop |
|  | Social validity | Ensure parents are aware of the programme as some parents might think we are teaching their children about sex. | Adapted - research team met with school leadership, school governing bodies and used community engagement strategies eg roadshows and radio |
|  | Delivery | They were concerns about the biomedical services that will be offered as nurses are thought of being not youth-friendly; they were also concerns about the times allocated for service delivery. | Adapted - all clinical staff were trained and time adjusted to suit most learners. |
