# Supplementary material for: Adaptation and Evaluation of a Multi‐Component School‐Based Sexual Reproductive Health Intervention Safer Choices Programme in Rural KwaZulu‐Natal, South Africa – A Process Evaluation
Source: J Int AIDS Soc. 2026 Oct 8;29(Suppl 5):e70192. doi: 10.1002/jia2.70192 (PMC13650518; doi:10.1002/jia2.70192)
Supplement: Supplementary file 3 [file JIA2-29-e70192-s003.docx]

**Appendix 2 - a. School schedule b. Checklist used to check completion of delivery and c. Debriefing form**

a. **Schedule**

| **School**  **Teacher in charge** | **Grade** | **Day of delivery** | **Period time** | **Comments** |
| --- | --- | --- | --- | --- |
| **School A**  *Ms A* | Grade 10 A  Grade 10 B  Grade 10 C  Grade 9 A  Grade 9 B | Tuesday  Tuesday  Tuesday  Friday  Friday | 11:30-12:30  13:30-14:30  09:45-10:45  09:45-10:45  07:45-08:45 |  |
| **School B**  *Mr B* | Grade 10  Grade 9 | Wednesday  Wednesday | 09:30 -10:30  13:15 – 14:15 |  |
| **School C**  *Ms C* | Grade 9  Grade 10 | Monday | 13:15-14:15 |  |
| **School D**  *Ms D* | Grade 10A and B  Grade 9 | Monday  Monday | 10:00  09:30 |  |
| **School E**  *Ms E* | Grade 9A  Grade 9B  Grade 10A, B, C | Monday  Monday  Monday | 07:50 – 08:30  12:15 -13:15  09:30-10:30 |  |

b. **Checklist of completion**

|  |  |  |  | **Lesson** | | | | | | | | | |  |
| --- | --- | --- | --- | --- | --- | --- | --- | --- | --- | --- | --- | --- | --- | --- |
| **School**  **Teacher in charge** | **Grade** | **Day of delivery** | **Period time** | **1**  Date  Done – Yes/No | **2**  Date  Done – Yes/No | **3**  Date  Done – Yes/No | **4**  Date  Done – Yes/No | **5**  Date  Done – Yes/No | **6**  Date  Done – Yes/No | **7**  Date  Done – Yes/No | **8**  Date  Done – Yes/No | **9**  Date  Done – Yes/No | **10**  Date  Done – Yes/No | **Comments** |
| **School A**  ***Ms A*** | **Grade 10 A**  **Grade 10 B**  **Grade 10 C**  **Grade 9 A**  **Grade 9 B** | **Tues**  **Tues**  **Tues**  **Frid**  **Frid** | **11:30-12:30**  **13:30-14:30**  **09:45-10:45**  **09:45-10:45**  **07:45-08:45** |  |  |  |  |  |  |  |  |  |  |  |
|  |  |  |  |  |  |  |  |  |  |  |  |  |  |  |

c. **Debriefing format**

School:

Confirm teams working at schools:

Progress to date:

Delivery Start date:

Delivery Completion date:

Which grades did you deliver to and how many lessons per grade or class you delivered (or the ones that you didn’t deliver and why?)

When did Intervention delivery strategy change (eg delivery per every LO lesson, combine classes, combine grades)? Which strategy was adopted and when?

Which strategy did you prefer and why?

Teacher involvement:

Challenges delivering Safer Choices? What can be changed?

Opportunities delivering Safer Choices? What can be changed?

What did you learn from the experience?

Comments:
